# Comparison of Maxillary Canine Retraction Using Split-Mouth Design With Dual Force Cuspid Retractor and T-loop Segmental Arch: A Split-Mouth Randomized Clinical Trial

**DOI:** 10.7759/cureus.35288

**Published:** 2023-02-22

**Authors:** Hemwati Nandan, Ch. Sudheer Kumar, Pragjyoti Jha

**Affiliations:** 1 Cleft and Craniofacial Orthodontics, GSR Institute of Craniofacial Surgery, Hyderabad, IND; 2 Department of Dentistry, Government Medical College, Siddipet, IND

**Keywords:** sectional mechanics, anchorage preservation, t-loop, dual force cuspid retractor, canine retraction, accelerated tooth movement

## Abstract

Introduction

This study was designed to explore the differences between two frictionless mechanics for canine retraction i.e., dual force cuspid retractor and T-loop segmental arch. T-loop for canine retraction creates a biomechanical system to deliver a predetermined force and a relatively constant moment-to-force ratio whereas dual force cuspid retractor uses power arms on buccal as well as palatal aspects for canine retraction. Bodily tooth movement can be achieved by both methods, but in this study, our main focus was to reduce the canine retraction timing with better three-dimensional control.

Method

This split-mouth study was conducted on a total of 20 cuspids of ten patients (five male and five female). Where one side of the arch was selected for T-loop and the other side for dual force cuspid retractor, randomly. Inclusion criteria for this study were; no congenitally missing teeth (excluding third molar), class I or class II molar relationship, no previous history of orthodontic treatment, good oral periodontal status, patients in whom extraction of maxillary first premolar during treatment was indicated. Both groups were compared for the duration of canine retraction, anchorage loss; tipping, and rotation of cuspid and molar, individually, after retraction.

Result

The result of this study showed that the duration of canine retraction was significantly less in group one, i.e., dual force cuspid retractor 73.8 ± 12.38 days, than in group two, i.e., T-loop 109.4 ± 16.71 days. The anchorage loss in group one was 0.60 ± 0.61 mm and that in group two was 2.40 ± 0.87 mm. Also, the amount of tipping and rotation of the cuspid and molar individually was significantly lesser in group one than in group two.

Conclusion

In this study, the dual force cuspid retractor shortens the duration of canine retraction with better three-dimensional control and better anchorage preservation when compared to T-loop.

## Introduction

To manage the arch-length tooth material discrepancy extraction of teeth or interproximal striping may be needed during orthodontic treatment. Patients with class II division 1 and bimaxillary protrusion require retraction, which can be done in one of two ways: A) en-masse retraction and B) two-step retraction. The two-step retraction includes canine retraction [1,2]. Closing the extraction space is a crucial aspect of orthodontic treatment. Different methods are used for canine retraction. Some of them are frictional (sliding), and others are non-fictional (segmental) [3]. Sliding mechanics take more time and require more anchorage. Proponents of the non-frictional approach claim to overcome these disadvantages [4]. Patients have always been very concerned about how long orthodontic treatment will take, especially in bicuspid extraction-based treatment plans where cuspid retraction requires a long time. Any options that shorten the duration of this stage will eventually assist in reducing the duration of the entire treatment. Acceleration of canine retraction helps in reducing the treatment timing for canine retraction. We can broadly divide it into four types: mechanical, pharmacological and hormonal, physical, and surgical. The change in the biomechanical system used may improve the speed of canine retraction [5-10].

In this study, the T-loop, first introduced by Burstone [11], and the dual force cuspid retractor, first described by Vyas et al. [4] were compared. Both methods exhibit features like the capability of cuspid retraction with anchorage preservation, axial inclination control, rotation control, and optimum biologic response. Therefore, this study was conducted to assess and contrast the aforementioned characteristics between these two approaches.

## Materials and methods

This split-mouth study was conducted on a total of 20 cuspids of ten patients (five males and five females). The institutional ethical committee approved the study. The study's IEC number is “No/TIDSHRC/Princi/20015/3489-A.” Each patient gave their signed, informed consent for this study. The inclusion criteria for this study are as follows: no congenitally missing teeth (excluding the third molar), class I or class II molar relationship, no previous history of orthodontic treatment, good oral periodontal status, and patients in whom extraction of maxillary first premolar during treatment was indicated.

Randomization was done with the online website www.randomization.com. All patients were divided into two groups using the fixed allocation randomization technique. Therefore, an equal number of canines were treated with either dual force cuspid retractor (group one) or T-loop (group two) strategies. Appliances were fabricated and inserted by a single practitioner for all the patients who were included in this split-mouth study. Assessments were conducted using an orthopantomogram, study models, and photographs.

Appliance fabrication and insertion

Dual Force Cuspid Retractor

**Figure 1:**
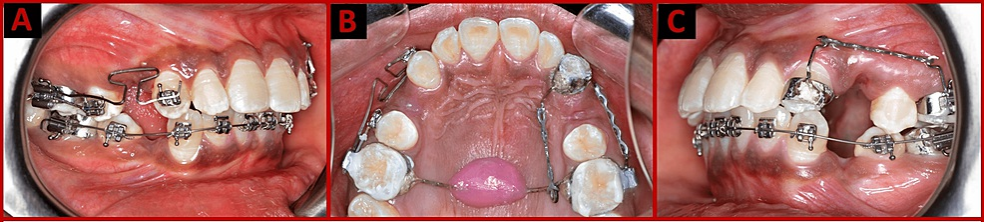
Appliance photographs: (A) T-loop, (B) Occlusal view, (C) Dual force cuspid retractor.

Dual force cuspid retractors were made with three components: molar band, power arm, and trans-palatal arch. In total, three power arms were needed: one for the molar on the buccal side, and two for the cuspid on the buccal and palatal sides. A 1.5 cm diameter of the nance palatal button and the palatal hook was added to the trans-palatal arch is depicted in Figure 1.

The bands were formed in the maxillary first molar and canine using 0.180 X 0.005 inch and 0.150 X 0.004 inch band material respectively. On the molar bands, a 0.022 x 0.028 inch upper triple molar tube was welded. The molar and canine bands were transferred to the impression, and then working models were produced to manufacture the device.

A 0.017 x 0.025-inch stainless steel wire was used to construct the power arms for the canine and molar teeth. Three power arms of 10 mm in length were fabricated maintaining a 2 mm gap from the gingival tissue, one for the molar and two for the canine. The molar power arm was cinched to the molar auxiliary tube and the canine power arms were soldered to the canine band (one on the buccal side and one on the palatal side).

**Figure 2:**
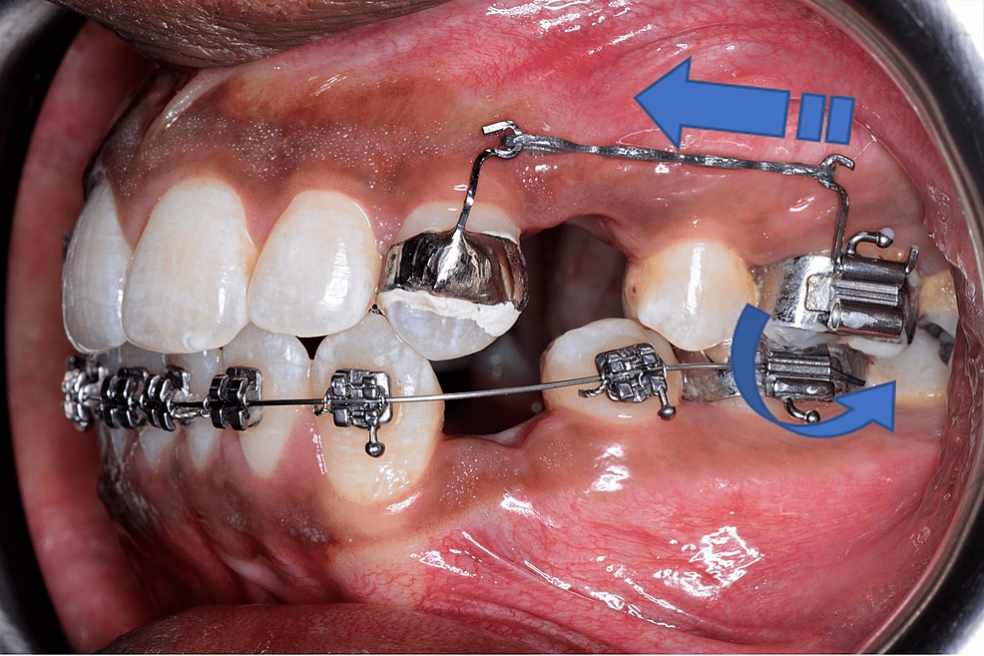
Mechanical advantage of power arm of molar

The trans-palatal arch was constructed using 1 mm hard round stainless-steel wire (Leone, liowire®) with the hook at the level of the palatal power arm of the cuspid. A nance palatal button of 1.5 cm diameter was built in the trans-palatal arch for further augmenting the anchorage. The trans-palatal arch and canine band with power arms were cemented. E-chain was then placed from the canine hook to the molar hook for cuspid retraction. The force was measured using a Dontrix gauge and a force of 150 grams was applied on the palatal as well as on the buccal aspect bilaterally, leading to a total force value of 300 grams. The mechanical advantage was provided by the elastic pull from the buccal power arm of the molar, which tends to distally tip the molar, augmenting the anchorage [4] as depicted in Figure 2.

T-loop

The T-loop is one of the most versatile devices for space closure. The T-loop was formed using 0.017 X 0.025-inch titanium molybdenum alloy (TMA) wire according to standard form and dimensions, as described by Burstone [11]. Before insertion of the T-loop, six pre-activation bends were given in the loop following which its trial activation was done and an anti-rotation bend was given. After this, the T-loop was inserted into the auxiliary tube of the molar and another end was pulled and inserted into the canine bracket slot. The total distal activation of the spring was 6 mm, which delivers an M/F ratio of 7:1 resulting in control tipping initially. As the space closes and the loop deactivates, the force delivered by it decreases. Translation occurs when the M/F ratio becomes 10:1. Further deactivation increases the M/F ratio to 12:1, which leads to root movement [12].

The distal tipping of the canines and the mesial tipping of the molars were determined by comparing and evaluating orthopantomogram x-rays taken before and after canine retraction. Study models were compared to evaluate canine and molar rotation as well as anchorage loss.

Orthopantomogram X-ray Analysis

**Figure 3:**
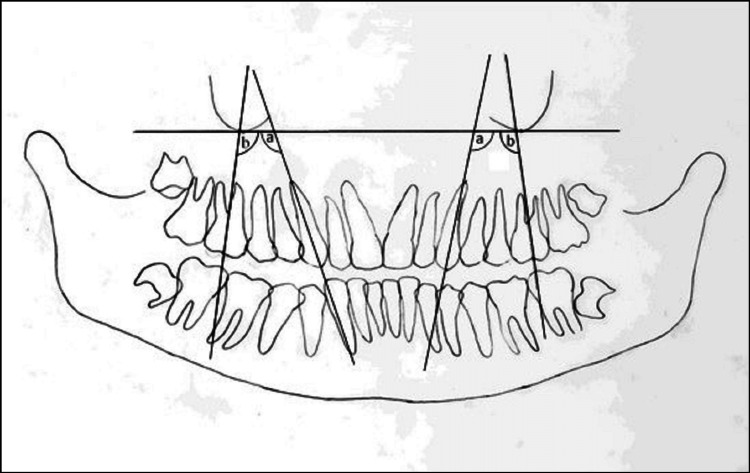
Orthopantomogram tracing showing: (A) Angulation of canine with orbital plane (B) Angulation of molar with orbital plane.

Change in angulation i.e. distal tipping of canine and mesial tipping of molars was assessed by measuring the angle formed by the long axis of canine and molar with the orbital plane towards the extraction site [13] as depicted in Figure 3.

Study Model Analysis

**Figure 4:**
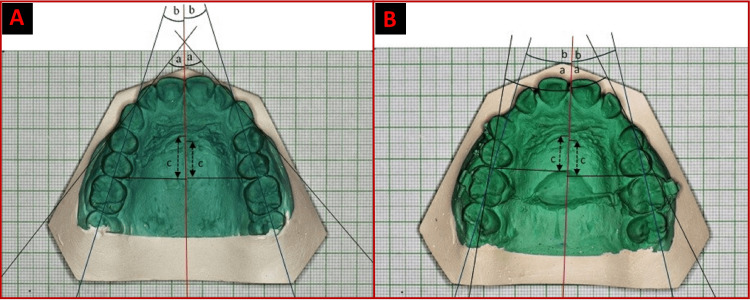
Study model analysis: (A) Pretreatment, (B) Posttreatment

The “Ziegler and Ingervall” method [14] was used to analyze the pre and post-retraction photographs of the study model. We utilized a digital single-lens reflex camera, a 1:1 macro lens with a 1.28 f, a telescoping tripod, and graph paper to prevent any errors or image magnification. Prior to printing, we cropped the image in a 3:2 ratio while keeping the 12-block graph length and eight-block graph width. The photo was then printed after being resized to 12 cm in length and 8 cm in width on a word document. We scale-measured the graph block to check for any magnification in the printed photograph. The angle formed by the line between the mesial and distal line angles of the cuspid and the mid-palatal raphe was measured to determine the canine's rotation. To track molar rotation, the angle between the distal and mesial contact points of the molar and mid-palatal raphe was measured. The distance between the third rugae and the perpendicular line from the mesial contact point of the molar to mid-palatal raphe was measured for determining anchorage loss [15] as depicted in Figure 4.

## Results

Statistical analysis

The recorded data from the orthopantomogram X-ray and study model analysis was compiled and entered in a spreadsheet computer program (Microsoft Excel 2007) and then exported to the data editor page of SPSS version 20.0 (SPSS Inc. Chicago Illinois, USA). Student t-tests and Paired t-tests were applied. The level of significance was set as p≤0.001 and p≤0.05. These analyses were obtained as depicted in Tables 1, 2.

**Table 1:** Duration of canine retraction and anchorage loss in Group-1 (Dual force cuspid retractor) and Group-2 (T-loop). Test applied: Student’s t-test for intergroup comparison; **p≤0.001 (Highly significant).

Variable	Group	Mean	Standard deviation	P value
Duration (in days)	Group-1	73.8 days	12.38	0.001^**^
Duration (in days)	Grour-2	109.4 days	16.71	0.001
Anchorage loss (in mm)	Group-1	0.60 mm	0.60	0.001^**^
Anchorage loss (in mm)	Group-2	2.40 mm	0.87	0.001

**Table 2:** Comparative evaluation of pre-treatment and post canine retraction; distal tipping of cuspid, mesial tipping of molar, rotation of cuspid and rotation of molar for both groups. Test applied: Paired t-test for intra-group comparison; *p≤0.05 (Significant), **p≤0.001 (Highly significant).

Analysis	Group	Variables	Mean difference	Standard deviation	p-value
Orthopantogram X-ray analysis	Group-1	Distal tipping of cuspid for Group-1 U3 To orbital plane (in degrees)	7.15^0^	5.84	0.004^*^
		Mesial tipping of molar for Group-1 U6 To orbital plane (in degrees)	2.25^0^	2.41	0.016^*^
	Group-2	Distal tipping of cuspid for Group-2 U3 to orbital plane (in degrees)	13.5^0^	7.07	0.001^**^
		Mesial tipping of molar for Group-2 U6 to orbital plane (in degrees)	7.2^0^	7.34	0.013^*^
Study Model Analysis	Group-1	Rotation of cuspid U3 to mid palatal raphe (in degrees) for Group-1	1.85^0^	1.35	0.002^*^
		Rotation of molar U6 to mid palatal raphe (in degrees) for Group-1	0.4^0^	1.9	0.537
	Group-2	Rotation of cuspid U3 to mid palatal raphe (in degrees) for Group-2	17.6^0^	8.91	0.001^**^
		Rotation of molar U6 to mid palatal raphe (in degrees) for Group-2	4.3^0^	3.88	0.007^*^

Results

This study was performed to compare the effectiveness of two frictionless mechanics for canine retraction. The appliance was inserted within 12 hours of extraction of the premolar to utilize the effect of the Regional acceleratory phenomenon. The appliance was in place till the completion of the canine retraction. Data obtained from this study for the individual groups are shown in [Appendixes 1, 2]. The following results were obtained after a comparative analysis of pre-treatment and post-retraction observations:

Canine retraction in group one was quicker compared to group two, and it took 73.8±12.38 days for the canine to fully retract while group two took 109.4±5.28 days. Anchorage loss, as the values of group one, was less than 0.60mm±0.60mm as compared to those of group two 0.87mm±0.27mm. Distal tipping of the canine in group one was less than 7.15±5.84 degrees as compared to that of group two 13.5±7.07 degrees. The mesial tipping of molars in group one was less than 2.25±2.41 degrees) as compared to that observed in group two 7.2+7.34 degrees. The canine rotation in group one was less 1.85±1.35 degrees compared to that observed in group two 17.6±8.91 degrees. The rotation of molars in group one was less 0.4±1.9 degrees as compared to that of group two 4.3±3.88 degrees.

## Discussion

The duration of orthodontic treatment has always been a concern for both the orthodontist as well as the patient. Longer duration of treatment has varied disadvantages like anchorage taxation, reduced patient compliance, and increased chances of periodontal damage. Many attempts have been made in the past to shorten the orthodontic treatment with the application of electric current, administration of prostaglandins, distraction osteogenesis, etc. The mechanics of canine retraction can be divided into two categories namely, friction mechanics and frictionless mechanics. Both techniques have advantages and disadvantages. Various canine retraction techniques are used in removable and fixed appliances. Tipping of canine and molar, mesial movement of anchor molar i.e., anchorage loss, rotation of canine, and creation of anterior deep bite are some of the side effects of the non-friction mechanics. A method to overcome the consequences of the traditional canine retraction technique is required. It has been demonstrated that more time is required to accomplish cuspid retraction with friction mechanics. Moreover, more force application is imperative as the majority of the force is consumed to overcome friction; thereby, ultimately reducing the available force for retraction. Hence, to prevent these consequences and enhance canine retraction, sectional mechanics for canine retraction was used. This study analyzed and compared the biomechanical efficacy of the dual force cuspid retractor and T-Loop.

Our research revealed that the dual force cuspid retractor group required less time for canine retraction. We believe this was due to the force applied from both the buccal and palatal aspects near the center of resistance, which led to less tipping of the tooth and more bodily movement of the tooth. However, in T-loop, the force was applied only from the buccal aspect, which led to initial tipping followed by translatory movement and then root up-righting; hence, taking more time.

In this study, there was less distal canine tipping in group one. According to Smith et al. [16] the force that passes through or near the center of resistance of the tooth causes less tipping and more bodily movement. This is seen with the dual force cuspid retractor, where the force for canine retraction passes nearer to the center of resistance of the tooth with the aid of power arms.

As per the literature, an optimum force of 300 grams can be applied for adequate canine retraction with a minimal change in axial molar inclination [17]. As the force for canine retraction was kept within the optimum limit, less mesial molar tipping and less anchorage loss were seen in group one in this study. The dual force cuspid retractor appliance administers force from both the labial and palatal sides for canine retraction, which appears to help control canine rotation when compared to methods that employ only buccal force.

In this study, the rate of canine retraction in group one was higher. We believe this is because the canine retraction received all of the applied force, with force coming from the buccal and palatal aspects close to the center of resistance. In group two, the force was applied only from the buccal aspect, which could result in canine rotation. To overcome this, an anti-rotation bend was placed in the T- Loop, which applied bucco-palatal force. This could be one of the reasons for the lower rate of retractions in this group.

The average amount of time needed for canine retraction in our study was 73.8 days in group one (0.69 mm/week), and 109.4 days in group two (0.47 mm/week). In our study canine retraction was relatively faster when compared to the canine retraction carried out by Samuels et al. [18] with the help of an e-module (0.19 mm/week) and coil spring (0.26 mm/week) as well as a study by Huffman et al. [19] where canine retraction was carried out using 0.016” wire (0.337 mm/week) and 0.020” wire (0.299 mm/week). Alfawal et al. B [9] evaluate the effect of piezocision and laser-assisted flapless corticotomy on canine retraction, the retraction rate is relatively slower and anchorage loss is similar as compared to our study. The canine retraction was relatively faster in our study when compared to canine retraction carried out by Hassan et al. [5] with self-ligating brackets and conventional-ligating brackets on 0.019 × 0.025˝ stainless archwire. A study by Al-Naoum et al. [10] on alveolar corticotomy accelerating orthodontic tooth movement and a study by, Jaber et al. [8] on laser-assisted flapless corticotomy for retracting upper canines with frictional mechanics. The retraction rate for this study was slightly higher for the first week when compared to our study. But second week onwards retraction was relatively slower in comparison to both types of frictionless methods used in our study. Abdul-Ela et al. [20] studies on maxillary canine retraction with corticotomy-facilitated orthodontics also showed a relatively slower rate in comparison to the result obtained by both types of frictionless methods used in our study. This slower retraction rate could be due to the force required to overcome the friction in their study; hence, requiring more time for retraction. This decreased rate of canine retraction may be caused by the force required to overcome the friction in their study, which lengthens the time needed for retraction. We began retraction within 12 hours of extraction, which caused a regional accelerating phenomenon and helped us shorten the length of time for canine retraction.

The dual force cuspid retractor group showed good control over the axial inclination of the canines as well. We think this is because the dual force cuspid retractor's appliance design, which applied less intermittent force and permitted proper axial control on the canine is to blame. Additionally, one of the explanations for the strong molar control we noticed in our study could be the trans-palatal arch with an acrylic button, which was used for anchorage strengthening.

The limitation of this study is that it only focuses on one jaw (maxilla) which limits the generalizability of the results. Evaluation of canine retraction in the lower arch was not feasible because of the absence of reliable and fixed landmarks. Blinding of the operator was not possible in this study, although blinding of the investigator was done by anonymized models and orthopantomogram to minimize possible bias. A skilled practitioner is required for appliance fabrication for segmented arch mechanics. This particular appliance design needed extra chair side time and lab assistance. The lateral incisor drifting during canine retraction contributes to reducing the overall treatment duration, but this has a temporary aesthetic effect.

## Conclusions

Frictionless mechanics carry out individual canine retraction in a more refined way and in less time when compared to friction mechanics. However, it requires precise skill for wire bending. In our research, we have seen that dual force cuspid retractor is biomechanically efficient, well accepted by patients, and demonstrates better three-dimensional control and better anchorage preservation. According to this research, dual force cuspid retractors can help to shorten the duration of orthodontic treatment, especially as cuspid retraction is achieved quicker with this method when compared to T-loops. In order to validate the research and learn more about the extra advantages and disadvantages of this kind of appliance, other practitioners must carry out research on larger samples.

## Appendices

Appendix 1 

**Table 3:** MASTER CHART (Group 1- DUAL FORCE CUSPID RETRACTOR) U3 to Orbital plane – Angulation of maxillary canine to orbital plane U6 to Orbital plane – Angulation of maxillary molar to orbital plane U3 to mid palatal Raphe – Angulation of maxillary canine to the mid-palatal raphae U6 to mid palatal Raphe - Angulation of maxillary molar to the mid-palatal raphae Distance between U6 and 3rd rugae - The distance between the 3rd rugae and perpendicular line from, mesial contact point of molar to mid palatal raphae

Patient		DURATION	ORTHOPANTOMOGRAPH		STUDY MODEL ANALYSIS				
No.			U3 to Orbital plane	U6 to Orbital plane	U3 to mid palatal Raphe	U6 to mid palatal Raphe	Distance between U6 and 3^rd ^rugae	Total retraction	Anchorage loss
	Pre Treatment	68 days	108.5^0^	97^0^	38^0^	13^0^	11mm	8mm	0mm
1	After Canine Retraction		90^0^	93^0^	33^0^	13^0^	11mm		
	Pre Treatment	96 days	100^0^	82.5^0^	21^0^	14^0^	9mm	8mm	2mm
2	After Canine Retraction		85.5^0^	81^0^	18.5^0^	11^0^	7mm		
	Pre Treatment	56 days	86^0^	85^0^	24^0^	16^0^	6.5mm	8mm	0.5mm
3	After Canine Retraction		85^0^	84^0^	21.5^0^	12^0^	6mm		
	Pre Treatment	88 days	100^0^	72^0^	37^0^	15^0^	5mm	7.5mm	0mm
4	After Canine Retraction		92^0^	73^0^	36^0^	16^0^	5mm		
	Pre Treatment	79 days	91.5^0^	90^0^	30^0^	11^0^	8mm	7mm	0.5mm
5	After Canine Retraction		82^0^	84^0^	29^0^	11^0^	7.5mm		
	Pre Treatment	74 days	92^0^	83^0^	19^0^	11^0^	9.5mm	7mm	1mm
6	After Canine Retraction		87^0^	77^0^	17^0^	13^0^	8.5mm		
	Pre Treatment	80 days	100^0^	97^0^	34^0^	22^0^	12mm	6.5mm	1mm
7	After Canine Retraction		97^0^	96^0^	33^0^	22^0^	11mm		
	Pre Treatment	65 days	86^0^	76^0^	37^0^	15^0^	7.5mm	7mm	0.5mm
8	After Canine Retraction		86^0^	73^0^	37^0^	15.5^0^	7mm		
	Pre Treatment	60 days	93^0^	78.5^0^	27^0^	13.5^0^	8.5mm	7mm	0.5mm
9	After Canine Retraction		85.5^0^	78.5^0^	25^0^	11.5^0^	8mm		
	Pre Treatment	72 days	91.5^0^	86.5^0^	28^0^	13^0^	7.5mm	7.5mm	0mm
10	After Canine Retraction		87^0^	85.5^0^	26.5^0^	14.5^0^	7.5mm		

Appendix 2 

**Table 4:** MASTER CHART (Group 2- “T-loop”) U3 to Orbital plane – Angulation of maxillary canine to orbital plane U6 to Orbital plane – Angulation of maxillary molar to orbital plane U3 to mid palatal Raphe – Angulation of maxillary canine to the mid-palatal raphae U6 to mid palatal Raphe - Angulation of maxillary molar to the mid-palatal raphae Distance between U6 and 3rd rugae - The distance between the 3rd rugae and perpendicular line from, mesial contact point of molar to mid palatal raphae

Patient		DURATION	ORTHOPANTOMOGRAPH		STUDY MODEL ANALYSIS				
No.			U3 to Orbital plane	U6 to Orbital plane	U3 to mid palatal Raphe	U6 to mid palatal Raphe	Distance between U6 and 3^rd ^rugae	Total retraction	Anchorage loss
	Pre Treatment	113 days	102^0^	97^0^	35^0^	15^0^	12mm	8mm	2mm
1	After Canine Retraction		82^0^	77^0^	11^0^	11.5^0^	10mm		
	Pre Treatment	139 days	100^0^	80^0^	20.5^0^	12^0^	11mm	8mm	4mm
2	After Canine Retraction		81^0^	81^0^	-8^0^	15^0^	7mm		
	Pre Treatment	85 days	86^0^	85^0^	37.5^0^	8.5^0^	8mm	7.5mm	1.5
3	After Canine Retraction		80^0^	78^0^	23^0^	4^0^	6.5mm		
	Pre Treatment	118 days	97^0^	82^0^	36^0^	15^0^	5.5mm	8mm	1mm
4	After Canine Retraction		80^0^	75^0^	9^0^	16^0^	4.5mm		
	Pre Treatment	113 days	81^0^	85^0^	18^0^	21^0^	8mm	7mm	3mm
5	After Canine Retraction		75^0^	81^0^	13^0^	15^0^	5mm		
	Pre Treatment	124 days	94.5^0^	87^0^	24^0^	14^0^	14mm	7mm	2.5mm
6	After Canine Retraction		70^0^	85^0^	-6^0^	6^0^	11.5mm		
	Pre Treatment	105 days	100^0^	98^0^	42^0^	29^0^	8.5mm	6.5mm	2mm
7	After Canine Retraction		83^0^	78^0^	32^0^	25^0^	6mm		
	Pre Treatment	92 days	86^0^	76^0^	41^0^	15^0^	10mm	7mm	3mm
8	After Canine Retraction		80^0^	70^0^	30^0^	11^0^	7mm		
	Pre Treatment	90 days	85.5^0^	83.5^0^	28^0^	14.5^0^	8mm	7mm	2mm
9	After Canine Retraction		80^0^	76.5^0^	14^0^	7^0^	6mm		
	Pre Treatment	115 days	86.5^0^	86^0^	30^0^	14.5^0^	10mm	7.5mm	3mm
10	After Canine Retraction		72.5^0^	86^0^	18^0^	5^0^	7mm		
